# Supplementary figures and images for: Biases in genome reconstruction from metagenomic data
Source: PeerJ. 2020 Oct 30;8:e10119. doi: 10.7717/peerj.10119 (PMC7605220; doi:10.7717/peerj.10119)

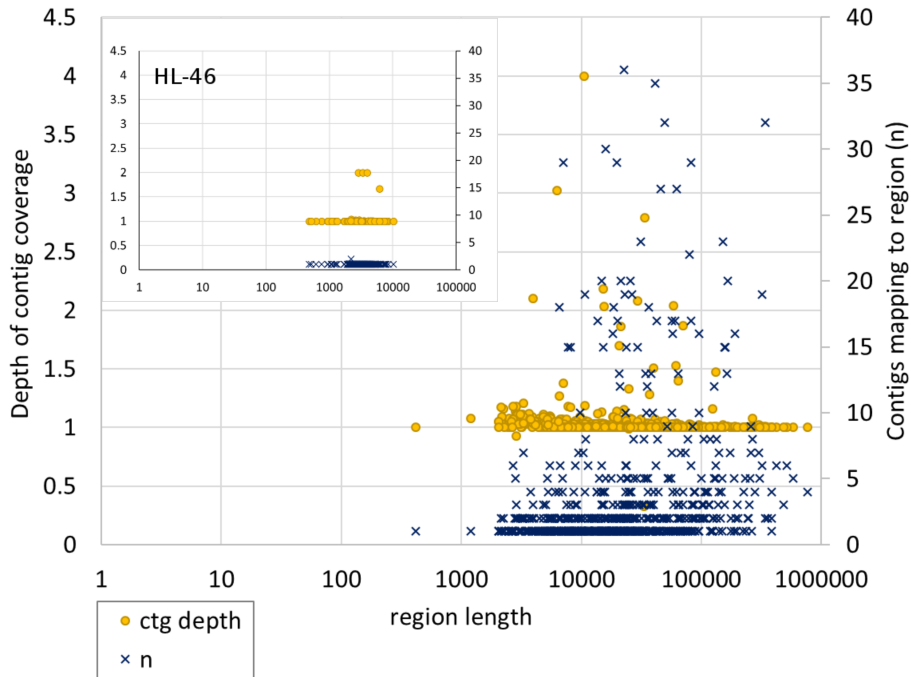

Supplement: Supplemental Information 1 — All contigs from the metagenome assembly set from which the MAGs were generated were searched against the CRs of each genome using NUCmer. The number of contigs mapping and the depth of coverage were determined for each CR and plotted by length. [file peerj-08-10119-s001.pdf]

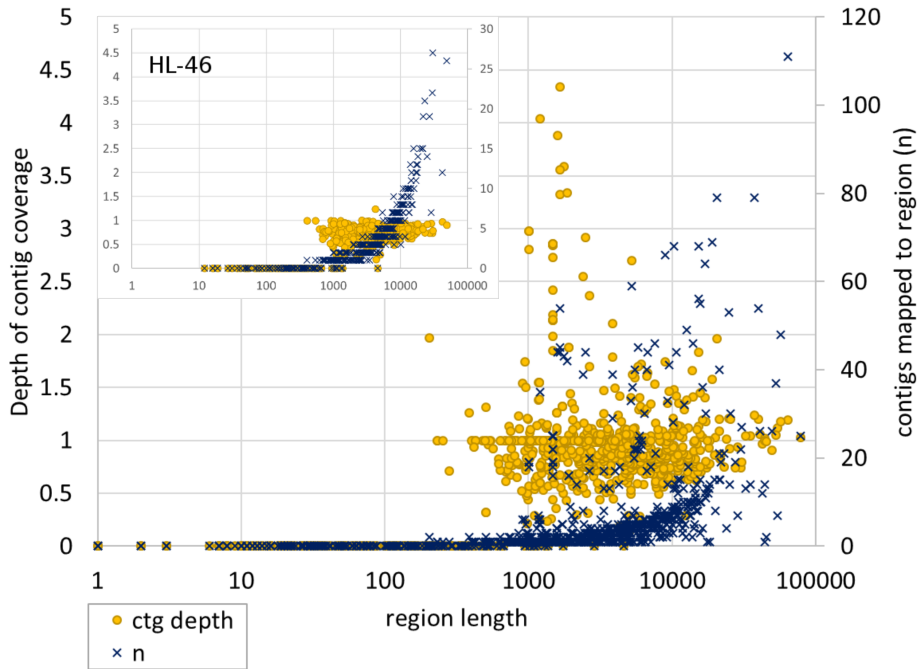

Supplement: Supplemental Information 2 — Analysis was performed as described for Figure S1, but on the NRs of each genome. [file peerj-08-10119-s002.pdf]

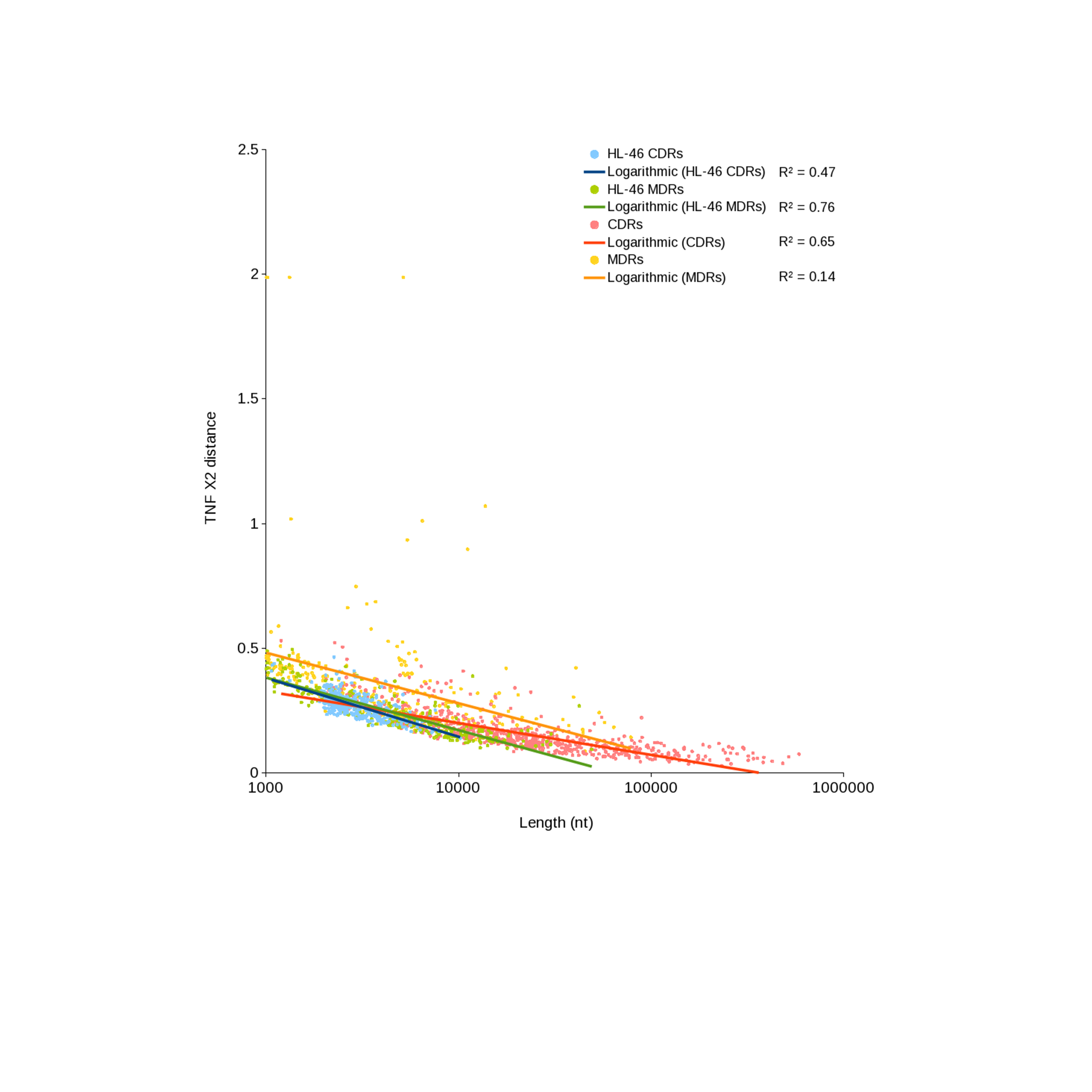

Supplement: Supplemental Information 3 — Comparison of tetranucleotide composition of CR and NR scaffolds as a function of scaffold length. Scaffolds from HL-46 serve as a reference standard because of their assumed random distribution. Logarithmic regressions and R2 values are presented. [file peerj-08-10119-s003.png]

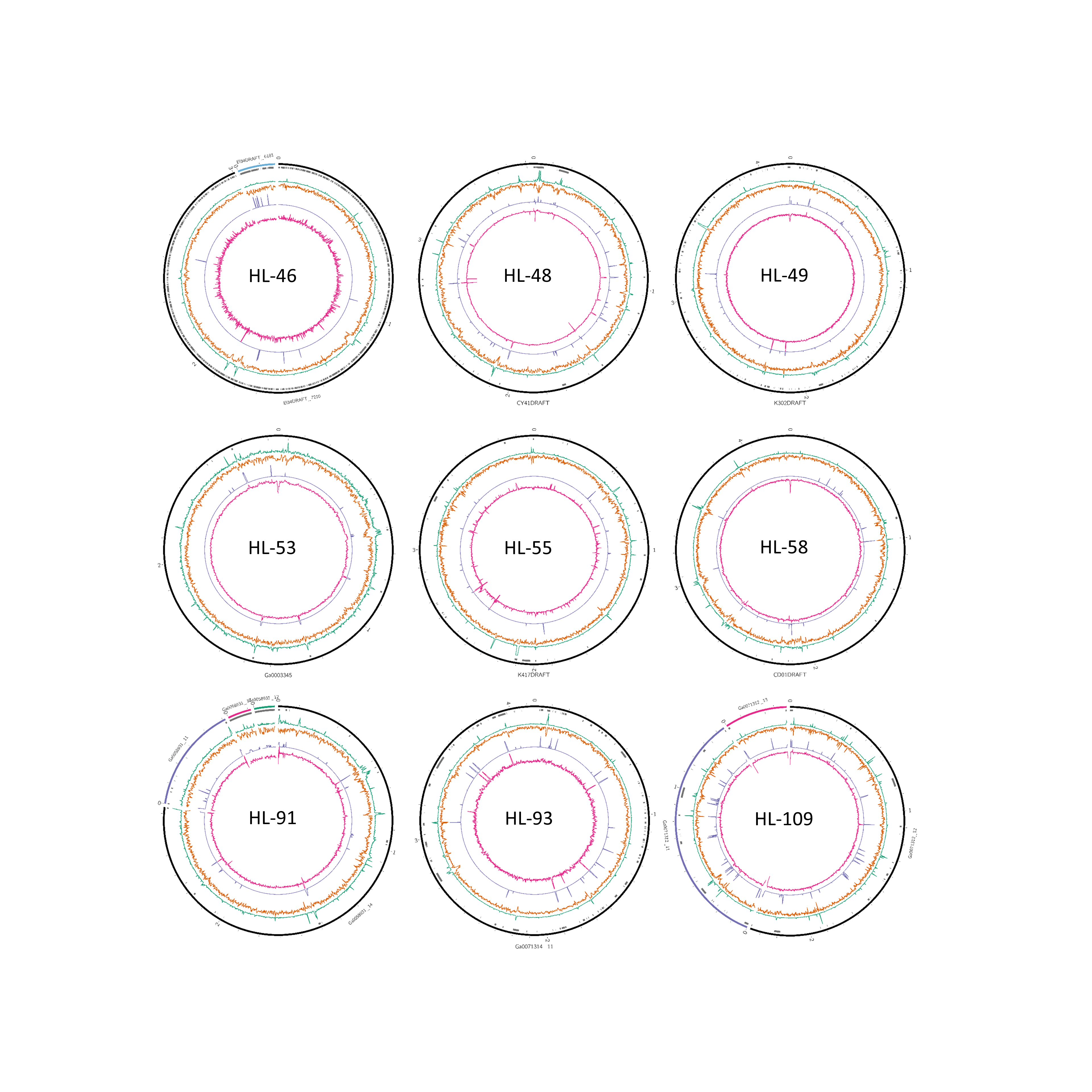

Supplement: Supplemental Information 4 — Data is presented as described in Figure 1 legend. [file peerj-08-10119-s004.png]
